# Is there any association between *Helicobacter pylori* and otitis media with effusion?^☆^

**DOI:** 10.1016/j.bjorl.2018.11.002

**Published:** 2018-12-19

**Authors:** Mohammad Ali Damghani, Elham Dehghan

**Affiliations:** Kerman University, Faculty of Medicine, Department of Otolaryngology, Kerman, Iran

**Keywords:** Otitis media with effusion, *Helicobacter pylori*, Polymerase chain reaction, Otite média com efusão, *Helicobacter pylori*, Reação em cadeia da polimerase

## Abstract

**Introduction:**

It is proposed that *Helicobacter pylori* can be responsible for the development of otitis media with effusion.

**Objective:**

The aim of this study is to investigate the prevalence of *H. pylori* in the adenoid tissue and fluid of the middle ear in patients who suffer from adenoid hyperplasia and otitis media with effusion in comparison with those who suffer from adenoid hyperplasia without otitis media with effusion.

**Methods:**

This is a case–control study that was carried out in 50 children of age 2–7 years old who were admitted with adenoid hyperplasia. Patients were divided into case and control groups. The study group included 25 patients with adenoid hyperplasia and otitis media with effusion and the control group included 25 patients with adenoid hyperplasia without otitis media with effusion. The patients in both groups underwent surgical adenoidectomy. For the case group we carried out myringotomy and placement of tympanostomy tube, and fluid samples were collected under sterile conditions. The samples were sent to the laboratory for polymerase chain reactions.

**Results:**

In the case group *H. pylori* was found to be positive in 18 samples of the middle ear fluid (70%) and in 1 polymerase chain reaction adenoid tissue sample (4%). In the control group *H. pylori* was positive in 3 samples of adenoid tissues (12%). There was no gender difference.

**Conclusion:**

*H. pylori* is one of the important bacteria that plays a role in the pathogenesis of otitis media with effusion. Whether adenoid tissue may be a reservoir for *H. Pylori* is unclear.

## Resumo

**Introdução:**

Propõe-se que o *Helicobacter pylori* possa ser responsável pelo desenvolvimento de otite média com efusão.

**Objetivo:**

Investigar a prevalência de *H. pylori* no tecido adenoideano e no fluido da orelha média em pacientes com hiperplasia de adenoide e otite média com efusão em comparação àqueles com hiperplasia de adenoide sem otite média com efusão.

**Método:**

Este é um estudo de caso-controle feito em 50 crianças de 2 a 7 anos de idade, com sinais e sintomas de hiperplasia de adenoide. Os pacientes foram divididos em grupo de estudo e grupo controle. O grupo de estudo incluiu 25 pacientes com hiperplasia de adenoide e otite média com efusão e o grupo controle incluiu 25 pacientes com hiperplasia de adenoide sem otite média com efusão. Os pacientes dos dois grupos foram submetidos a adenoidectomia e, no grupo de estudo, realizou-se também miringotomia com colocação de tubo de ventilação e amostras de fluidos foram coletadas sob condições estéreis. As amostras foram enviadas para o laboratório, para investigação por reação de polimerase em cadeia.

**Resultados:**

No grupo de estudo, houve positividade para *H. pylori* em 18 amostras do fluido de orelha média (70%) e uma amostra de tecido adenoideano foi positiva na reação de polimerase em cadeia (4%). No grupo controle, houve positividade para *H. pylori* em 3 amostras de tecido adenoideano (12%). Não houve diferença entre os gêneros.

**Conclusão:**

*H. pylori* é uma das bactérias importantes que desempenham um papel na patogênese da otite médica com efusão. Se o tecido adenoideano pode ou não representar um reservatório para *H. pylori* ainda necessita ser esclarecido.

## Introduction

Otitis media with effusion (OME) is defined as middle ear effusion without signs and symptoms of acute inflammation found in acute otitis media.^1^ The pathophysiology of the disease is multifactorial, including allergy, autoimmunity, gastroesophageal reflux disease, bacteria, viral and eustachian tube dysfunction. From ears with chronic OME, *Haemophilus influenzae* was the single most common pathogen, and other common bacteria included *Streptococcus pneumonia* and *Moraxella catarrhalis*. Other bacteria account for a small percentage of cases.^2^ Recent studies have investigated the possibility of a relationship between OME and the presence of *Helicobacter pylori* (*H. pylori*) in the middle ear.^3^ By the age of 10 years approximately 75% of children are infected with *H. pylori*. Many body regions other than the gastrointestinal tract have been investigated for the presence of this microorganism. In recent years, *H. pylori* has been found in adenoid tissue, nasal polyp tissue and the middle ear.^4^ *H. pylori* has also been shown to be present in high ratios in nasal polyps, laryngeal samples, and vocal cord lesions using real-time polymerase chain reaction (PCR) method.^5^, ^6^ One potential cause of OME is the reflux of gastric contents into the region of the nasopharyngeal mucosa, which initiates an inflammatory process.^7^ This pathophysiological mechanism has been frequently questioned in recent study.^8^, ^9^ Animal studies have shown that reflux leads to eustachian tube dysfunction. The eustachian tube is immature and its angle is wider in children. Therefore gastric contents can more easily reach the middle ear and induce an inflammatory process.^10^ Few studies have investigated the relationship between OME, pepsinogen levels and *H. pylori* presence separately.^11^ Several methods are used for the detection of *H. pylori.* It has been shown that PCR method has almost 100% sensitivity and specificity in the detection, identification and quantitation of *H. pylori* in biological samples.^12^ The aim of this study is to investigate the presence of *H. pylori* in adenoid tissue of patients with adenoid hyperplasia without OME in comparison with patients who have adenoid hyperplasia with OME.

## Methods

Our study is a case–control study that was performed on 50 children between the ages 2 and 7 years old with adenoid hyperplasia, with median age of 4.5 years, who were admitted to the ENT department of Shafa hospital (Kerman, Iran) between October 2013 and March 2015. The patients were enrolled in the study by non-random easy sampling method.

Inclusion criteria were signs and symptoms of adenoid hyperplasia that were confirmed by lateral neck radiography in the extension position. The patients have been divided into two groups of case and control, based on the presence or absence of signs and symptoms of OME. After history taking and clinical examination including otoscopy, diagnosis of OME was confirmed as well as tympanometry for confirmation of OME. The patients in the case group had OME but the patients in the control group did not.

Exclusion criteria included patients who did not consent to enter the study, a history of previous adenoidectomy, neurological disorders, genetic syndromes, craniofacial abnormalities such as Down syndrome, and other causes of airway obstruction such as deviated septum, nasal polyps, and turbinate hypertrophy. In addition patients with active infection were excluded. We described objectives and methods of the study to parents of the patients and they were asked questions to ensure that they understood the procedure. Afterwards, they were allowed to ask their possible questions. Later, written permissions were obtained from all parents. Then all patients underwent surgical adenoidectomy under the same conditions, and adenoid samples were prepared with curette. A core biopsy specimen was taken from each adenoid tissue; collected samples were placed immediately in PSB buffer. In addition, for the case group, samples from the middle ear effusion were taken. For this purpose, outer ear canal was cleaned with 70% alcohol solution, then an incision was made at the anterior inferior quadrant of the eardrum. Samples were collected from middle ear fluid and were placed in the PSB transmission medium. The effusion and adenoid tissue samples were transported to microbiology laboratory. The samples were frozen immediately after removal and stored at −20 °C; when required, specimens were thawed, and supernatants of centrifuged specimens were assayed. To measure the DNA of *H. pylori*, genomic isolation kit was used.

Measurement was performed by a spectrophotometer. Thus the existence of the DNA of *H. pylori* was determined in the bands of 260–280 nm by absorption rate. At the end, all patients’ information was entered the checklist. Data was reported by descriptive statistics and analysis was done by chi-square and Fisher exact tests and SPSS software was applied.

The ethical code of this article is IR.KMU.REC.1395.344.

## Results

**Figure 1:**
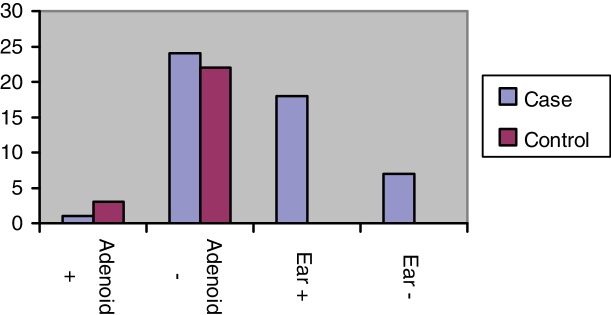
The frequency of *H. pylori* in adenoids and middle ear effusions of case and control groups.

**Figure 2:**
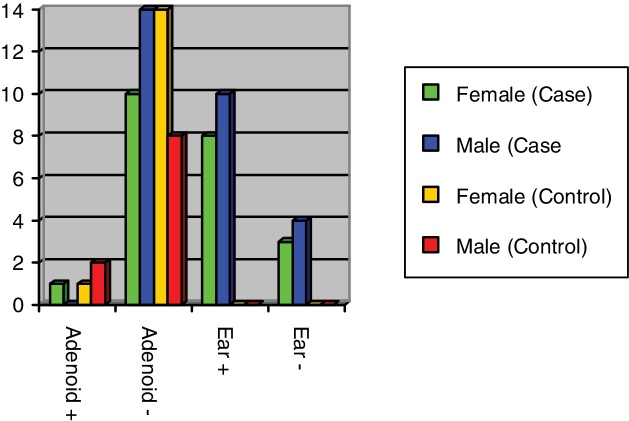
*H. pylori* frequency based on gender in case and control groups.

The number of patients who entered the study was 50 children who were studied in two groups of 25 cases and 25 controls. 24 patients (48%) were male and 26 (52%) were female. Out of 50 patients, 18 patients (70%), in the case group had *H. pylori* in the middle ear fluid, 3 patients (12%) in the control group had *H. pylori* in adenoid tissue and 1 patient (4%), in the case group had *H. pylori* in adenoid tissue. Thus the frequency of *H. pylori* in the middle ear fluid in the case group is more than it's frequency in adenoid tissues of both case and control groups (Table 1 and Fig. 1). Out of 26 girls, two patients (7.7%) (one patient in the case group and one patient in the control group), had *H. pylori* in adenoid tissue. Out of 24 boys, two (8.3%) in the control group had *H. pylori* in adenoid tissue and none of the boys in case group had bacteria in adenoid tissue. Eight girls (30.8%) in the case group had *H. pylori* in the middle ear fluid samples and 10 boys (41.7%) in the case group had *H. pylori* in the middle ear fluid. Results are reported in Table 2. So according to Table 2 we can say there is no significant gender difference in our comparison (Fig. 2).

**Table 1:** *Helicobacter pylori* (*H. pylori*) positivity in case and control groups and comparison between groups, by chi-square test.

Groups	Control		Case			
	Adenoid		Adenoid		Middle ear fluid	
Number of patients	25					
*H. pylori* presence	Present	Absent	Present	Absent	Present	Absent
Detection rate of *H. pylori*	3 (12%)	22 (88%)	1 (4%)	24 (96%)	18 (72%)	7 (28%)

*p*-Value of comparing *H. pylori* in adenoid tissues of the case and control groups is 0.609.

*p*-Value of comparing *H. pylori* in adenoid tissue and middle ear effusion of the case group is 0.000.

*p*-Value of comparing *H. pylori* in adenoid tissue of the control group and middle ear effusion of the case group is 0.001.

**Table 2:** The relationship between *Helicobacter pylori* (*H. pylori*) and gender among control and case study groups done by fisher's exact test.

Group	Gender	*H. pylori* presence in adenoid		*p*-Value
		Negative	Positive	
Case	Female	1 (9.1%)	10 (90.9%)	0.44
	Male	0 (0%)	14 (100%)	

Control	Female	1 (6.7%)	14 (93.3%)	0.54
	Male	2 (20%)	8 (80%)	

Total	Female	2 (7.7%)	24 (92.3%)	1
	Male	2 (8.3%)	22 (91.7%)

## Discussion

OME is one of the most common causes of hearing loss among children in developed countries. OME is characterized by fluid accumulation in the middle ear with no active infection; if the disease persists for more than three months, it is called chronic OME. In several studies, the relationship between *H. pylori* and the pathogenesis of various upper aerodigestive tract problems have been shown. *H. pylori* for reproduction needs a microaerophilic environment. This environment is present in the middle ear of OME patients. The mechanism of the colonization of these bacteria in the gastric mucosa is still unclear. It is said this bacteria grows by implanting inside the mucosal epithelium of the stomach, protected by impermeable mucus layer to the gastric acid. The PH of the luminal side of the mucus layer is 1.0–2.0 and the PH of the mucosal side is approximately 7.4. The PH of the middle ear effusions is also between 7.0–9.0. In *H. pylori* infections mucosal metaplasia and goblet cell hyperplasia occurs, a similar situation in OME.^7^, ^13^ In this study, we applied PCR for the detection of *H. pylori* by specimen collection from middle ear. We calculated *H. pylori* positivity rate not on the basis of the number of ears, but the number of patients because we would like to know the existence rate of *H. pylori* in each individual. We found *H. pylori* in 70% of the middle ear effusions is positive, and this is relatively high in comparison with other studies such as Chul Von et al.^14^ (the prevalence of *H. pylori* was 30%), so we suggest that *H. pylori* could be an important etiologic factor for development of OME. Agirdir et al.^15^ reported that *H. pylori* positivity was 66.6% in effusion of 30 patients with OME and the adenoid of the patient group and the control group showed no significant difference in the prevalence of *H. pylori.* Yilmase et al.^10^ reported that the PCR of middle ear effusion was positive for *H. pylori* in 45% of the patients with OME. In another study by Yilmaz et al.^16^ eighteen subjects with OME and adenoid hyperplasia and 20 subjects with only adenoid hyperplasia were compared. The results showed that in the study group, *H. pylori* was positive in 67% of children. None of the tissue samples obtained from adenoids of study group and only one of the tissue samples in the control group was positive with PCR. There are two possible explanations for why *H. pylori* has been discovered in the effusion of the middle ear. The first is that the tonsil and adenoid act as a reservoir for *H. pylori.* Since the tonsil, adenoid, and Eustachian tube are anatomically close, this can allow for *H. pylori* to spread directly.^17^, ^18^ The second possibility is due to gastroesophageal reflux, since the gastric fluid can affect the middle ear through the Eustachian tube, and *H. pylori* that is mixed in with the gastric fluid can then be detected in the middle ear. There is an association between chronic middle ear problems and gastroesophageal reflux. Higher concentration of pepsin/pepsinogen in the middle ear than serum of patients with OME has been reported by Tasker et al.^7^

In a study that published in 2008 by Fancy et al.^3^ they reported, in comparison of 45 patients with adenoid hyperplasia and OME with 35 patients who just had adenoid hyperplasia, there was no significant difference in the incidence of this infection between the two groups.

In another study by Saki et al.^19^ the prevalence of *H. pylori* in patients with OME was studied. Eighty-four patients who were subjected to adenoidectomy and myringotomy were included in the study group. Ninety-one patients who had only adenoidectomy were selected as the control group. Adenoid samples were positive for *H. pylori* in 25% patients in the study group and 19.8% patients in the control group. In the study group, 42.8% effusion samples (Otitis Media) of the patients were positive for *H. pylori.* They showed that the colonization of *H. pylori* in adenoid tissue and in the middle ear may be involved in the pathogenesis of OME.

In the present study, we found that the presence of *H. pylori* in adenoid samples of study group was 4% and in control group was 12%; this finding is not significant in comparison of *H. pylori* positivity of patients with OME that was 70% indicating that adenoid tissue is not a reservoir for *H. pylori*. We hope future studies will be done on the relationship between OME and *H. pylori* in the world population.

## Conclusion

Our study showed that there is significant *H. pylori* presence in middle ear of the children with chronic OME, indicating *H. pylori* have a possible role in OME pathogenesis. In addition we detected *H. pylori* presence in 4 of 50 adenoid specimens, supporting the idea that adenoid tissue does not act as a reservoir for *H. pylori*.

## Conflicts of interest

The authors declare no conflicts of interest.
